# The Impact of Pleural Effusion on Long-Term Mortality in Patients Undergoing Transcatheter Aortic Valve Implantation

**DOI:** 10.3390/jcm14051596

**Published:** 2025-02-26

**Authors:** Fatma Esin, Hakan Bozkurt, Berkay Palac, Bahadır Akar, Tuncay Kiris, Emre Özdemir, Mustafa Karaca

**Affiliations:** Department of Cardiology, Atatürk Training and Research Hospital, Izmir Katip Çelebi University, Izmir 35360, Turkey; dr.fatmakesin@gmail.com (F.E.); hakanbozkurt12@outlook.com (H.B.); bpalac35@gmail.com (B.P.); bakar1998@hotmail.com (B.A.); emreozdemir27@yahoo.com.tr (E.Ö.); mustafakaraca99@hotmail.com (M.K.)

**Keywords:** pleural effusion, transcatheter aortic valve implantation, long-term mortality

## Abstract

**Background/Objectives**: Pleural effusions may be seen in patients with severe AS complicated by hemodynamically significant heart failure. However, there are no data on the association between pleural effusion and long-term mortality in patients undergoing transcatheter aortic valve implantation (TAVI). This study aimed to assess the impact of pre-procedural pleural effusion on long-term mortality in these patients. **Methods**: A retrospective, single-center analysis was conducted on 401 patients who underwent TAVI between January 2010 and December 2023. The patients were categorized into two groups based on the presence of pleural effusion, which was assessed via pre-procedural imaging using thoracic computed tomography (CT). **Results**: Pleural effusion was present in 158 patients (39.4%). The patients with pleural effusion had significantly higher long-term mortality rates compared to those without pleural effusion (46.2% vs. 24.3%, *p* < 0.001). Multivariate analysis identified pleural effusion as an independent predictor of long-term mortality (HR: 1.568, 95% CI: 1.065–2.308, *p* = 0.023). Also, the patients with pleural effusions had a higher long-term mortality rate compared with those without pleural effusions (log-rank *p* < 0.001). **Conclusions**: Pre-procedural pleural effusion is independently associated with increased long-term mortality in TAVI patients. Early recognition and management of pleural effusion are critical for optimizing outcomes in this high-risk population.

## 1. Introduction

Transcatheter aortic valve implantation (TAVI) has revolutionized the treatment of severe symptomatic aortic stenosis (AS), particularly in patients who are at high or prohibitive risk for surgical valve replacement. This minimally invasive procedure offers significant benefits, including reduced perioperative morbidity and faster recovery compared to traditional surgical approaches, and has become a preferred option for many patients with advanced aortic valve disease [1,2]. However, despite these advancements, identifying factors that influence long-term outcomes after TAVI remains critical for optimizing patient care [3].

Pleural effusion, defined as the accumulation of fluid within the pleural cavity, is a frequent finding in patients with cardiovascular diseases, particularly those with heart failure. The mechanisms underlying pleural effusion in these patients are multifactorial, often involving systemic venous congestion due to elevated right heart pressures, hypoalbuminemia, and a reduced glomerular filtration rate (GFR) [4,5]. Elevated left ventricular (LV) filling pressures also play a significant role, as increased pulmonary venous pressures can result in fluid transuding into the pleural space [6]. Hypoalbuminemia further exacerbates fluid retention by reducing oncotic pressure, promoting the formation and persistence of pleural effusion [7]. Despite these observations, the specific impact of pre-procedural pleural effusion on long-term mortality in TAVI patients remains underexplored. Understanding this relationship is crucial, as it could aid in risk stratification and the development of perioperative management strategies, ultimately improving patient outcomes [8,9].

This study aimed to evaluate the association between pre-procedural pleural effusion and long-term mortality in patients undergoing TAVI.

## 2. Materials and Methods

### 2.1. Patients, Study Design, and Data Collection

This retrospective, single-center study was conducted at a tertiary-level education and research hospital from January 2010 to December 2023. This study included adult patients (aged ≥ 18 years) with severe symptomatic aortic stenosis who underwent TAVI during the specified period. Patients were eligible if they had complete pre-procedural imaging, including thoracic computed tomography (CT), transthoracic echocardiography (TTE), coronary angiography (CAG), and clinical records. Data were extracted from the hospital’s electronic medical records and included demographic characteristics, clinical parameters, and laboratory values. The patients were stratified into two groups based on the presence or absence of pre-procedural pleural effusion. Baseline characteristics, procedural outcomes, and follow-up data were compared between the two groups.

### 2.2. Inclusion and Exclusion Criteria

The exclusion criteria were as follows: patients with incomplete or poor-quality imaging data or missing clinical or follow-up records. This study was conducted according to the guidelines of the Declaration of Helsinki and was approved by the Institutional Review Board (or Ethics Committee) of Izmir Katip Çelebi University, Atatürk Training and Research Hospital (2024/0341). Due to this study’s retrospective nature, the need for informed consent was waived by the Medical Ethics Committee of Izmir Katip Çelebi University, Atatürk Training and Research Hospital.

### 2.3. Imaging and Assessments

Transthoracic echocardiographic (TTE) examinations were performed using a Philips EPIQ 7 ultrasound system (Philips Healthcare, Amsterdam, The Netherlands). Comprehensive TTE was performed to evaluate aortic valve morphology and function, as well as left ventricular parameters. Coronary angiography was performed prior to TAVI to assess coronary artery disease (CAD) using a Siemens Artis zee angiography system (Siemens Healthineers, Erlangen, Germany). Pre-procedural pleural effusion was assessed through CT scans performed using a GE Revolution EVO 128-slice CT scanner (GE Healthcare, Chicago, IL, USA) as part of a routine preoperative evaluation. Pleural effusion was categorized as present or absent based on the CT findings.

### 2.4. Study Endpoints

The primary outcome was long-term mortality, defined as death from any cause during the follow-up period.

### 2.5. Statistical Analysis

Continuous variables were summarized as means ± standard deviations or medians with interquartile ranges based on their distributions. Categorical variables were expressed as frequencies and percentages. Comparisons between groups were performed using Student’s *t*-test or the Mann–Whitney U test for continuous variables and the chi-square or Fisher’s exact tests for categorical variables.

Kaplan–Meier survival curves were generated to evaluate long-term mortality, and differences between groups were assessed using the log-rank test. A multivariable Cox proportional hazards regression was performed to identify independent predictors of long-term mortality, adjusting for potential confounders. Variables with *p*-values < 0.10 in the univariate analysis were included in the multivariable model. Statistical significance was set at *p* < 0.05. All statistical analyses were conducted using SPSS version 26 (SPSS Inc., Chicago, IL, USA), and R software (version R 4.1.2) was used for advanced modeling and visualization.

## 3. Results

### 3.1. Patient Characteristics

This study included 401 patients who underwent TAVI. They were divided into two groups based on the presence of pre-procedural pleural effusion: 243 patients without pleural effusion and 158 patients with pleural effusion. The patients with pleural effusion were slightly older than those without (77.3 ± 8.4 vs. 76.1 ± 7.7 years; *p* = 0.118). Male patients were more prevalent in the group without pleural effusion (55.3% vs. 44.7%; *p* = 0.034).

The prevalence of diabetes mellitus (DM), hypertension (HT), dyslipidemia, carotid artery disease, smoking, and CAD was similar between the groups (*p* > 0.05). In contrast, chronic kidney disease (CKD) was significantly more common in the patients with pleural effusion (39% vs. 19%; *p* < 0.001), as was heart failure (39.2% vs. 18.9%; *p* < 0.001). There were no significant differences in body mass index (BMI), history of heart valve surgery, peripheral artery disease (PAD), chronic obstructive pulmonary disease (COPD), prior stroke, atrial fibrillation (AF), or prior percutaneous coronary intervention (PCI) between the groups (*p* > 0.05). These findings are summarized in Table 1.

Patients with pleural effusion had significantly lower GFR (54.40 ± 20.21 vs. 62.29 ± 17.25 mL/min; *p* < 0.001) and albumin levels (33.94 ± 5.01 vs. 36.76 ± 6.52 g/dL; *p* = 0.037). Other laboratory parameters, including hemoglobin (HGB), the platelet count (PLT), and the white blood cell count (WBC), were comparable between the groups (*p* > 0.05).

The echocardiographic assessments revealed that the patients with pleural effusion had a significantly lower left ventricular ejection fraction (LVEF) (49.7 ± 12.5% vs. 56.6 ± 8.6%; *p* < 0.001). Moderate-to-severe mitral regurgitation (MR) and tricuspid regurgitation (TR) were more frequent in the pleural effusion group (56.3% vs. 33.3%, *p* < 0.001 for MR; 44.3% vs. 21.8%, *p* < 0.001 for TR). Additionally, the systolic pulmonary artery pressure (SPAP) was significantly higher in the patients with pleural effusion (44.3 ± 15.6 mmHg vs. 36.0 ± 12.4 mmHg; *p* < 0.001). Aortic valve gradients, including the peak gradient (AV PG) and mean gradient (AV MG), did not differ significantly between the groups (*p* > 0.05). These findings are presented in Table 2.

### 3.2. Prognostic Impact of Pleural Effusion in Patients Who Underwent TAVI

The patients with pleural effusion had a higher mortality rate compared with those without pleural effusion during long-term follow-up (46% vs. 24%, *p* < 0.001, Table 1).

Univariate analysis revealed that the presence of pleural effusion was associated with a two-fold increase in mortality risk (HR: 2.104, 95% CI: 1.485–2.979, *p* < 0.001, Table 3). This association persisted in the multivariate model, where pleural effusion was confirmed as an independent predictor of mortality (HR: 1.568, 95% CI: 1.065–2.308, *p* = 0.023, Table 3). Other independent predictors of mortality included advanced age (HR: 1.031, 95% CI: 1.004–1.058, *p* = 0.026), a reduced GFR (HR: 0.986, 95% CI: 0.976–0.996, *p* = 0.009), and an elevated SPAP (HR: 1.019, 95% CI: 1.003–1.035, *p* = 0.026). These results are detailed in Table 3.

### 3.3. Survival Analysis

**Figure 1:**
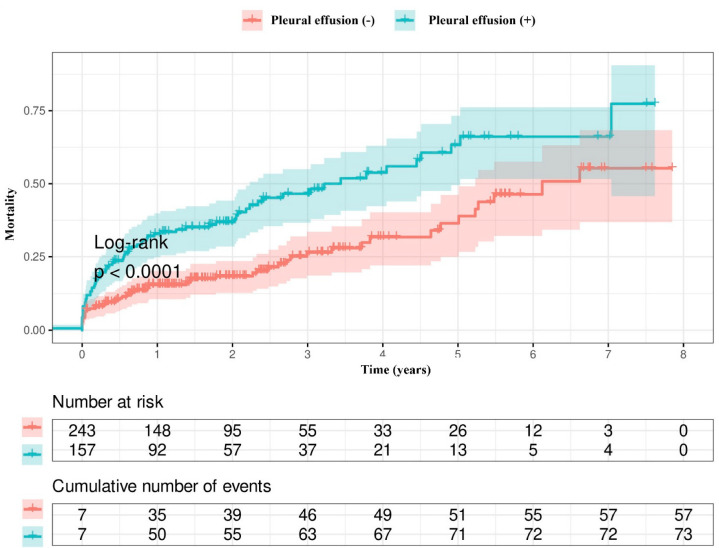
The mortality rates according to the presence or absence of pleural effusion.

**Figure 2:**
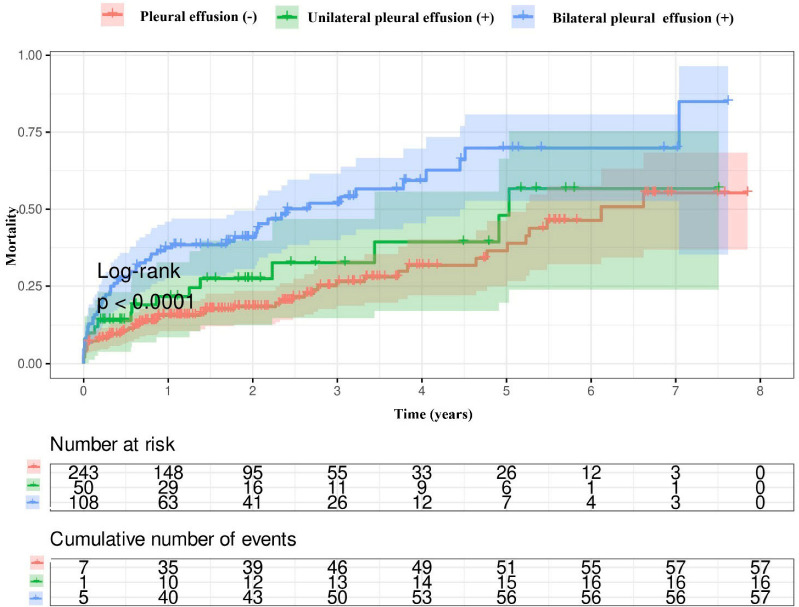
The mortality rates according to the size of the effusion.

**Figure 3:**
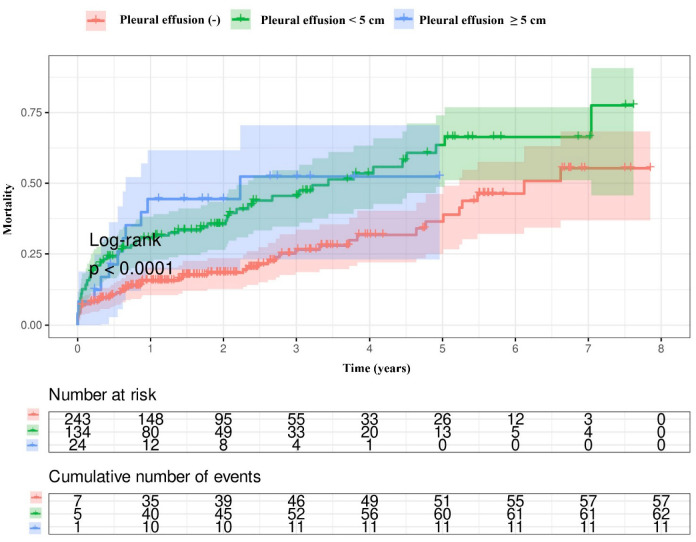
The mortality rates according to whether the effusion was unilateral or bilateral.

Kaplan–Meier survival curves showed that the patients with pleural effusion had a higher mortality rate compared with those without (Figure 1). Also, a higher mortality rate was observed in patients with bilateral pleural effusion than in both patients with unilateral pleural effusion and those without pleural effusion (Figure 2). There was no difference in the mortality rates between patients with effusion sizes ≥ 5 cm and patients with effusion sizes < 5 cm (Figure 3).

## 4. Discussion

Our study identified pre-procedural pleural effusion as an independent predictor of long-term mortality in TAVI patients. Patients with pleural effusion showed significantly higher mortality rates compared to those without effusion, even after adjusting for confounders.

Pleural effusion, observed in 20% to 40% of TAVI patients [10,11], often reflects systemic congestion due to elevated cardiac filling pressures and impaired lymphatic drainage in advanced heart failure and chronic kidney disease [12,13]. Systemic inflammation and hypoalbuminemia also contribute by increasing vascular permeability and reducing oncotic pressure, respectively [14,15]. These mechanisms underscore pleural effusion as a marker of systemic and cardiac decompensation, highlighting its prognostic value. An elevated SPAP contributes to pulmonary venous congestion, leading to fluid accumulation in the pleural space [16]. Similarly, MR and TR exacerbate atrial pressures, further promoting effusion formation [17,18]. Previous reports have shown that late-onset pacemaker-related pleural effusions due to improper pacing settings are rarely seen after pacemaker implantation, especially in elderly patients [19,20]. Patients experiencing this may develop rapid or slow accumulation and reaccumulation of fluid in the pleural space [19,20].

Several studies support the link between pleural effusion and mortality across clinical settings. Schiefenhövel et al. reported significantly increased mortality rates in cardiac surgery patients with pleural effusion [10]. Das et al. emphasized its role as a marker of systemic congestion and advanced heart failure [13]. Bediwy et al. highlighted that pleural effusion often coexists with markers of organ dysfunction and poor survival outcomes in critically ill patients [21]. Collectively, these findings underscore the importance of recognizing and managing pleural effusion in high-risk populations.

Pleural effusion was observed as a critical finding in our cohort and was strongly associated with increased mortality. This observation aligns with prior studies, including a study by Breuss et al., who demonstrated that bilateral pleural effusion in severe aortic stenosis (AS) is a marker of adverse hemodynamic conditions, including elevated pulmonary artery wedge pressure and a reduced stroke volume index, and is associated with a 2.7-fold increase in post-aortic valve replacement (AVR) mortality [22]. Elevated SPAP was identified as another significant predictor in our study, which corroborated findings by Tang et al., who linked pulmonary hypertension to poor outcomes in TAVI populations [23].

A reduced GFR was also a significant risk factor, aligning with the findings of D’Errigo et al., who highlighted the adverse impact of renal impairment on survival in TAVI patients [24]. CKD exacerbates systemic congestion and inflammation, complicating recovery. Advanced age, an elevated SPAP, and a reduced GFR were all significantly associated with long-term mortality in our TAVI cohort. These findings are further supported by the findings of Kjønås et al. [25].

Advanced age, as highlighted in prior studies, adds complexity to procedural risk, particularly in the presence of significant comorbidities [26]. Nombela-Franco et al. emphasized the negative prognostic impact of valvular dysfunction, particularly moderate-to-severe TR and MR, on survival in TAVI populations [27]. In the presented study, although moderate-to-severe TR and MR were independently associated with mortality in the univariate analysis, they did not reach statistical significance in the multivariate analysis. The interplay of these factors underscores the importance of individualized risk stratification and a multidisciplinary approach to care.

Valve types, including balloon-expandable and self-expanding models, were analyzed in our study. While no significant differences in outcomes were noted, Makkar et al. suggest that valve selection should be tailored to individual anatomical and clinical factors to optimize procedural success [28]. Balloon-expandable valves offer precise deployment in challenging anatomies, whereas self-expanding valves are advantageous in patients with larger annular sizes.

Our findings highlight the multifactorial nature of mortality in TAVI patients. Pleural effusion, SPAP elevation, a reduced GFR, and valvular dysfunction are significant contributors. Previous studies have also shown that both exercise stress echocardiography (ESE) and speckle-tracking echocardiography (STE) analysis are useful for risk assessment in patients with aortic stenosis [29,30,31,32]. Studies incorporating these advanced imaging techniques and hemodynamic assessment are needed to refine risk stratification and improve long-term outcomes in these patients. These findings emphasize the need for multidisciplinary management strategies to address the complex interplay of comorbidities in this high-risk population.

## 5. Study Limitations

This study has several limitations. First, its retrospective nature may have introduced selection bias and limited the establishment of causal relationships. Second, the absence of direct hemodynamic catheterization data restricted precise assessment of pre-procedural pressures, and this study relied on echocardiographic surrogates such as the SPAP and LVEF. Third, the relatively small sample size may have reduced the statistical power, particularly for identifying associations with less common predictors. Fourth, the lack of long-term follow-up data on functional outcomes and quality of life limited our understanding of the broader implications of our findings. Fifth, our single-center design may affect the generalizability of the results, as procedural protocols and patient characteristics could vary across institutions.

Finally, although we accounted for key comorbidities, the absence of frailty indices and biomarkers may underestimate the multifactorial complexity of TAVI patient risk profiles. Future prospective, multicenter studies with larger cohorts and comprehensive data collection are warranted to address these gaps.

## 6. Conclusions

Pre-procedural pleural effusion was a significant predictor of long-term mortality in TAVI patients. These findings emphasize the need for comprehensive pre-procedural evaluations and tailored multidisciplinary strategies to improve patient outcomes.

## Figures and Tables

**Table 1:** Demographic and clinical characteristics of TAVI patients grouped according to pleural effusion status.

Characteristics (*n*, %)	No Pleural Effusion (*n* = 243)	Pleural Effusion Present (*n* = 158)	*p*-Value
Age (years)	76.1 ± 7.7	77.3 ± 8.4	0.118
Gender (male)	109 (55.3%)	88 (44.7%)	0.034
DM	87 (64.4%)	48 (35.6%)	0.262
HT	167 (62.3%)	101 (37.7%)	0.318
Dyslipidemia	156 (58.0%)	113 (42.0%)	0.127
Carotid artery disease	3 (1.2%)	4 (2.5%)	0.332
Smoking	7 (2.9%)	6 (3.8%)	0.612
CAD	200 (82.3%)	133 (84.2%)	0.625
History of heart valve surgery	7 (2.9%)	2 (1.3%)	0.286
PAD	28 (11.5%)	28 (17.7%)	0.080
Heart failure	46 (18.9%)	62 (39.2%)	<0.001
COPD	23 (9.5%)	12 (7.6%)	0.517
Previous stroke	17 (7.0%)	12 (7.6%)	0.821
CKD, *n* (%)	47 (19)	61 (39)	<0.001
Previous AF	58 (23.9%)	44 (27.8%)	0.371
PCI before TAVI	21 (8.6%)	19 (12.0%)	0.269
LVEF (%)	56.6 ± 8.6	49.7 ± 12.5	<0.001
MR (moderate/severe)	81 (33.3%)	89 (56.3%)	<0.001
TR (moderate/severe)	53 (21.8%)	70 (44.3%)	<0.001
Valve type, *n* (%)			0.278
Balloon-expandable	86 (36)	48 (31)	
Self-expanding	154 (64)	109 (69)	
TPM post-TAVI	98 (40.3%)	68 (43.0%)	0.389
PPM post-TAVI	40 (16.5%)	30 (19.0%)	0.257
Complications after TAVI, *n* (%)			0.409
Pseudoaneurysm	2 (1)	1(1)	
Stent placement for arterial injury	10 (4)	9 (6)	
Surgical repair of arterial injury	6 (3)	8 (5)	
Cardiac tamponade after TAVI	3 (3)	5 (3)	
Long-term mortality, *n* (%)	57 (24)	73 (46)	<0.001

Abbreviations: TAVI, transcatheter aortic valve implantation; CAD, coronary artery disease; DM, diabetes mellitus; HT, hypertension; PAD, peripheral artery disease; COPD, chronic obstructive pulmonary disease; CKD, chronic kidney disease; AF, atrial fibrillation; LVEF, left ventricular ejection fraction; MR, mitral regurgitation (moderate/severe); TR, tricuspid regurgitation (moderate/severe); TPM, Temporary Pacemaker; PPM, Permanent Pacemaker.

**Table 2:** Laboratory findings in TAVI patients grouped according to the presence of pleural effusion.

Parameters	No Pleural Effusion (*n* = 243)	Pleural Effusion Present (*n* = 158)	*p*-Value
HGB (g/dL)	11.3 ± 1.7	10.8 ± 1.8	0.005
PLT (×10^3^/μL)	217.8 ± 81.3	218.0 ± 87.1	0.681
GFR (mL/min)	62.29 ± 17.25	54.40 ± 20.21	<0.001
Albumin (g/dL)	36.76 ± 6.52	33.94 ± 5.01	0.037
WBC (×10^3^/μL)	7.85 ± 3.31	8.38 ± 3.48	0.13
BMI (kg/m^2^)	30.38 ± 5.16	27.31 ± 6.01	0.214
AV PG (mmHg)	76.33 ± 21.85	75.79 ± 24.32	0.817
AV MG (mmHg)	46.45 ± 14.36	45.86 ± 15.99	0.702
SPAP (mmHg)	36.0 ± 12.4	44.3 ± 15.6	<0.001

Abbreviations: HGB: hemoglobin; PLT: platelet count; GFR: glomerular filtration rate; WBC: white blood cell count; BMI: body mass index; AV PG: Peak Gradient Across Aortic Valve; AV MG: Mean Gradient Across Aortic Valve; SPAP: systolic pulmonary artery pressure.

**Table 3:** Predictors of mortality in univariate and multivariate analyses.

Variables	Univariate			Multivariate		
	HR	95% CI	*p*	HR	95% CI	*p*
Age	1.043	1.017–1.067	0.001	1.031	1.004–1.058	0.026
GFR	0.979	0.970–0.988	<0.001	0.986	0.976–0.996	0.009
MR (moderate/severe)	1.468	1.038–2.077	0.030			
TR (moderate/severe)	1.471	1.031–2.101	0.034			
Balloon-expandable vs. self-expanding	0.632	0.388–1.029	0.065			
SPAP	1.022	1.010–1.034	<0.001	1.019	1.003–1.035	0.026
Pleural effusion	2.104	1.485–2.979	<0.001	1.568	1.065–2.308	0.023

Abbreviations: HR: Hazard Ratio; CI: Confidence Interval; GFR: glomerular filtration rate; MR: mitral regurgitation; TR: tricuspid regurgitation; SPAP: systolic pulmonary artery pressure.

## Data Availability

The data supporting this study’s findings are available from the corresponding author upon reasonable request.
